# Successful use of extended cardiopulmonary resuscitation followed by extracorporeal oxygenation after venlafaxine-induced takotsubo cardiomyopathy and cardiac arrest: a case report

**DOI:** 10.1186/s13256-021-03031-w

**Published:** 2021-09-27

**Authors:** Sune Forsberg, Lis Abazi, Pär Forsman

**Affiliations:** 1 Department of Anaesthesiology and Intensive Care, Norrtälje Hospital, Norrtälje, Sweden; 2 Centre for Resuscitation Science, Department of Medicine, Karolinska Institutet, Stockholm, Sweden; 3 Swedish Poisons Information Centre, Stockholm, Sweden; 4 ECMO Centre Karolinska, Karolinska University Hospital, Stockholm, Sweden

**Keywords:** Cardiac arrest, Intoxication, ECMO, CPR

## Abstract

**Background:**

Severe venlafaxine intoxication may cause arrhythmias, cardiac failure, and even cardiac arrest.

**Case presentation:**

A 48-year-old caucasian male with an extensive psychiatric history ingested a high dose of venlafaxine causing a serum venlafaxine concentration of 12.6 mg/L 24 hours after ingestion. Seven hours post-ingestion, he experienced tonic–clonic seizures, and 8 hours later, takotsubo cardiomyopathy was recognized followed by cardiac arrest. The patient was resuscitated with prolonged cardiopulmonary resuscitation including ongoing automatic external compressions during helicopter transportation to a tertiary hospital for extracorporeal membrane oxygenation treatment. Despite a cardiopulmonary resuscitation duration of 2 hours, 36 hours of extracorporeal membrane oxygenation, and a total of 30 days of intensive care, the patient made a full recovery.

**Conclusion:**

In cases of intoxication-induced cardiac arrests among otherwise young and healthy patients, prolonged cardiopulmonary resuscitation and extracorporeal circulation can be a life-saving bridge to recovery.

## Background

The antidepressant venlafaxine is a serotonin, a norepinephrine, and to some extent a dopamine reuptake inhibitor. In severe cases, the most severe complications of overdoses are seizures, cardiac dysfunction, and death [1]. Takotsubo cardiomyopathy (TTC) is reported after the ongoing intake of prescribed doses of venlafaxine as well as after acute overdoses [2, 3].

## Case presentation

A 48-year-old caucasian male with an extensive psychiatric history of depression was admitted to the intensive care unit at a local hospital following intoxication of approximately 20 g venlafaxine, 450 mg zolpidem, and 250 mg propiomazine. His past history included intensive care hospitalization due to two suicidal attempts through pharmacological intoxication and self-harm with a knife. On admission, he was awake but drowsy and had a Glasgow Coma Scale (GCS) 13 of 15 (E3 + V4 + M6) but stable respiratory and circulatory status. His electrocardiography (ECG) was normal with a sinus rhythm of 57 beats per minute. Laboratory findings were within normal range (Table 1). Admission occurred approximately 2 hours after intake of the pills. Activated charcoal suspension was administered via a nasogastric tube upon arrival at the intensive care unit.

**Table 1:**
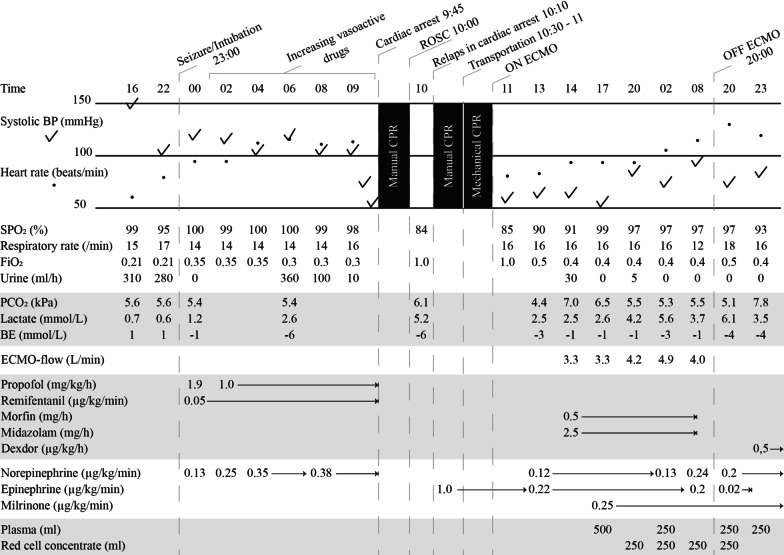
Clinical data of the case day 1–3

Time intervals differ during the described period.

The patient was stable during the first 5 hours at the intensive care unit (ICU), but then 7 hours post-intoxication he experienced tonic–clonic seizures. This was successfully treated with diazepam 10 mg intravenously, and he was subsequently intubated and sedated with propofol and remifentanil. Vasopressor support with low-dose norepinephrine was also initiated.

Nine hours after ingestion, his circulation began to slowly fail. Increased doses of norepinephrine were required, and the patient developed metabolic acidosis with lactate elevation. Fifteen hours after ingestion, the patient’s blood pressure fell rapidly despite high doses of norepinephrine. Prompt echocardiography revealed prominent hypokinesia with akinesia from the mid-left ventricle to the apex, as in TTC, with a left ventricle ejection fraction (EF) of 20%. The right ventricle was also affected with a tricuspid annular plane systolic excursion (TAPSE) measuring 10 mm. At this point, dobutamine was added to norepinephrine, but only a few micrograms were infused prior to cardiac arrest.

Shortly after the addition of dobutamine, his circulatory status deteriorated further, and the patient suffered a cardiac arrest with an initial rhythm of asystole. Advanced cardiac life support was initiated according to national guidelines including manual CPR and intravenous epinephrine. The patient was given three doses of epinephrine 1 mg intravenously, and after 5 minutes without return of spontaneous circulation, the ECMO center at a tertiary hospital was contacted. Fifteen minutes post cardiac arrest, the patient regained circulation temporarily for approximately 10 minutes before relapsing into cardiac arrest. The patient’s circulatory status and blood pressure were, however, inadequate during these 10 minutes. High-dose epinephrine infusion (1.0 µg/kg/minute) was initiated and manual CPR continued for another 30 minutes. The patient was then transported by helicopter to a tertiary hospital using mechanical CPR (AutoPulse Resuscitation System) and ongoing epinephrine infusion. Transportation time was approximately 30 minutes. One hour 15 minutes after the cardiac arrest, the patient was delivered to the tertiary hospital for ECMO initiation.

Upon arrival at the tertiary hospital and prior to initiation of ECMO, the patient had a sinus rhythm but very low cardiac output. Blood pressure was 55/45 mmHg without cardiac arrest, and external compression was continued whilst ECMO system treatment was established. Short pauses were taken during specifically vulnerable periods of the cannulation procedure. Lactate peaked at 4.6 mmol/L. Two hours after the cardiac arrest, the patient was on ECMO.

After ECMO initiation treatment with sedation and circulatory drugs continued, continuous renal replacement therapy (CRRT) was initiated. CRRT was initiated due to anuria and creatinine level of 265 µmol/L. Sedation was reinitiated using midazolam and morphine instead of propofol and remifentanil. The amount of epinephrine was significantly decreased during the initial hours of ECMO treatment, and norepinephrine and milrinone were used instead. Multiple plasma and red cell concentrate (RCC) transfusion were also required due to significant bleeding from the femoral artery catheter. The patient was successfully weaned after 32 hours of ECMO. Epinephrine infusion was terminated the same day, while milrinone continued until the following day.

The patient was transported back to the primary hospital with decreasing doses of norepinephrine the day after ECMO termination. Three days after the cardiac arrest, his cardiac function was echodynamically restored with an EF of above 55%. Values of the cardiac biomarker NT-proBNP decreased from 8360 ng/L the day after the cardiac arrest to 1190 ng/L 36 days after the intoxication. He was ventilated for 8 days and received CRRT for 3 weeks. Two days after extubation, the patient gradually regained consciousness. Thirty days after the intoxication, he had regained normal cardiac and cognitive function and left the hospital for further psychiatric treatment. The patient’s renal function was fully restored with normal creatinine level (82 µmol/L) 7 weeks after the intoxication. He was finally discharged in good health without his former prescribed psychiatric medications. Two years later, his cardiac and renal function were normal, although his psychiatric medication was reinstated.

The serum venlafaxine concentration 24 hours after ingestion was 12.6 mg/L, but this laboratory result was not received until 1 week after ingestion.

## Discussion

This is a case report where a lethal dose of venlafaxine [1, 4] led to a sustained cardiac arrest that was successfully treated with ECMO. As venlafaxine has a half-life of approximately 15 hours in overdose cases, the patient’s venlafaxine concentration of 12.6 mg/L 24 hours after ingestion indicates a significantly higher peak concentration would have been reached [5]. In line with previous reported cases, this concentration level is comfortably within the concentration range that can cause severe failure of ventricular dysfunction as well as conduction abnormalities [1, 4, 6, 7].

Conduction abnormalities due to overdose of venlafaxine are believed to be caused by sodium channel dysfunction. However, ECG examination in this case did not show any significant arrhythmias prior to the cardiac arrest, and effects on sodium channel function could not be observed due to the high concentration of venlafaxine [8, 9].

Severe left ventricular dysfunction was, however, observed. There has been speculation that the cause of TTC is an increase in catecholamines from venlafaxine [10]. While the exact causes of TTC are unknown, multiple studies show that levels of catecholamines are increased both systemically and locally in the myocardium [8]. Such increase in catecholamines causes myocardial and microvascular dysfunction with coronary spasm, which is considered a potential cause of TTC. This is believed to be mediated by sympathetic innervation in the myocardium, resulting in toxic overload that leads to myocardial dysfunction, and by systemically increased catecholamines through the adrenal medulla.

In this case, we did not suspect the patient’s condition to progress to circulatory failure and TTC. Echocardiography was not performed until 1 hour prior to the cardiac arrest when the patients’ blood pressure began to fail and lactate levels increased despite increased treatment with norepinephrine. With better knowledge of the clinical course of high-dose intoxication of venlafaxine, cardiac complications could have been suspected earlier. Subsequently, a more informed choice of treatment, inotropy versus vasopressor, could have been made at an earlier stage. Despite this, even when TTC has been found at an early stage, the treatment of choice is far from clear. Hypotension needs to be treated in order to preserve coronary perfusion and mitigate left ventricular failure. If vasopressors are chosen, then an increase in afterload may aggravate the ventricular failure. If inotropes are chosen, both inotropy and tachyarrhythmias may worsen the left ventricular outflow tract obstruction that can often be found in TTC [11]. Treatment choice must, therefore, be individualized, and catecholamines should be used with caution. In cases when pharmacological treatment is insufficient, treatment should be complemented with mechanical support such as ECMO.

Our case illustrates that successful outcome can be achieved even in situations where the patient’s condition deteriorates to cardiac arrest and pharmacological treatment is insufficient. Through good cardiac resuscitation and the use of automatic external compressions, which are known to be as effective as manual compressions, it is possible to maintain sufficient circulation [12]. Given the prolonged half-life of venlafaxine, the heart’s recovery from severe failure takes additional time, and therefore, a temporary rescue circulation with ECMO was preferable despite the 2 hours separating the primary hospital from the ECMO center. It is therefore important to consider cardiac support (such as ECMO) as a bridge to recovery, especially for young and otherwise healthy patients even in cases of prolonged CPR. This bridge may be a life-saving measure even when a patient has a seemingly unfavorable prognosis.

## Conclusions

In cases of overdose-induced cardiac arrests, with prolonged periods of CPR, it is important to consider extracorporeal circulation as a bridge to spontaneous recovery.

## Data Availability

All relevant data generated during this work are included in this published article, and further data can be requested from the corresponding author.

## Abbreviations

CPR: Cardiopulmonary resuscitation
ECG: Electrocardiography
ECMO: Extracorporeal membrane oxygenation
GCS: Glasgow Coma Scale
NT-proNP: N-terminal pro-B-type natriuretic peptide
RCC: Red cell concentrate
TTC: Takotsubo cardiomyopathy
